# Polyphenols Influence the Development of Endometrial Cancer by Modulating the Gut Microbiota

**DOI:** 10.3390/nu16050681

**Published:** 2024-02-28

**Authors:** Ewa Baranowska-Wójcik, Anna Winiarska-Mieczan, Piotr Olcha, Małgorzata Kwiecień, Karolina Jachimowicz-Rogowska, Łukasz Nowakowski, Andrzej Miturski, Krzysztof Gałczyński

**Affiliations:** 1 Department of Biotechnology, Microbiology and Human Nutrition, University of Life Sciences, Skromna Street 8, 20-704 Lublin, Poland; ewa.baranowska@up.lublin.pl; 2 Institute of Animal Nutrition and Bromatology, Department of Bromatology and Nutrition Physiology, University of Life Sciences in Lublin, Akademicka 13, 20-950 Lublin, Poland; malgorzata.kwiecien@up.lublin.pl (M.K.); karolina.jachimowicz@up.lublin.pl (K.J.-R.); 3 Department of Gynecology and Gynecological Endocrinology, Medical University of Lublin, Aleje Racławickie 23, 20-049 Lublin, Poland; piotrolcha@op.pl; 4 Department of Gynecology, 1st Clinical Military Hospital in Lublin, Al. Raclawickie 23, 20-049 Lublin, Poland; luknowakow@gmail.com (Ł.N.); a.miturski@gmail.com (A.M.); 5 Faculty of Medical Sciences and Health Sciences, Siedlce University of Natural Sciences and Humanities, Konarskiego 2, 08-110 Siedlce, Poland; krzysztof.galczynski@gmail.com

**Keywords:** prebiotics, polyphenols, gynaecological tumours, endometrial cancer, gut microbiota

## Abstract

Dysbiosis of the microbiota in the gastrointestinal tract can induce the development of gynaecological tumours, particularly in postmenopausal women, by causing DNA damage and alterations in metabolite metabolism. Dysbiosis also complicates cancer treatment by influencing the body’s immune response and disrupting the sensitivity to chemotherapy drugs. Therefore, it is crucial to maintain homeostasis in the gut microbiota through the effective use of food components that affect its structure. Recent studies have shown that polyphenols, which are likely to be the most important secondary metabolites produced by plants, exhibit prebiotic properties. They affect the structure of the gut microbiota and the synthesis of metabolites. In this review, we summarise the current state of knowledge, focusing on the impact of polyphenols on the development of gynaecological tumours, particularly endometrial cancer, and emphasising that polyphenol consumption leads to beneficial modifications in the structure of the gut microbiota.

## 1. Introduction

Diverse microorganisms inhabit the human intestine, including archaea, bacteria, viruses, and fungi [1]. The microbiome comprises approximately 1500 species of bacteria [2]. In healthy individuals, the gut flora exhibits a certain degree of similarity and stability, playing a role in the metabolism, immune response, and physiological function of key organs. However, dysbiosis of the gut microbiota can affect human health, including the development of malignant gynaecological tumours, particularly in postmenopausal women [3]. Dysbiosis also hinders cancer treatment by influencing the body’s immune response and disrupting the sensitivity to chemotherapy drugs [4]. Therefore, maintaining homeostasis of the gut microbiota is of utmost importance, including the effective use of food components that influence its structure.

The primary goal of supplementing the diet with probiotics and prebiotics is to increase the population of beneficial bacteria, eliminate pathogens, and induce lasting changes in the composition of gut flora [5]. Alleviating gut dysbiosis is associated with reduced autoimmune responses, decreased inflammation, and improved intestinal integrity by enhancing protein expression in the intestinal epithelium [6]. Probiotic microorganisms are lactic acid bacteria belonging to the *Firmicutes phylum*, *Bacilli class*, and *Lactobacillales* order, encompassing over 50 genera from 6 families and more than 300 species [7]. Probiotics can inhibit inflammation and oxidative stress by suppressing lipid peroxidation, modulating the secretion of pro-inflammatory and anti-inflammatory cytokines, and maintaining antioxidant enzyme activity [5]. Prebiotics, on the other hand, are food components that positively impact well-being and health by selectively stimulating the growth and/or activity of gut microbiota, thereby also positively influencing the gut structure [8]. This group includes indigestible oligosaccharides, and their positive effects on gut microbiota structure and health have been demonstrated in numerous studies [2,9,10]. However, recent research has shown that polyphenols, which are likely the most important secondary metabolites produced by plants, also exhibit prebiotic properties. They possess strong antioxidant and anti-inflammatory properties [11]. The prebiotic properties of polyphenols stem from the gut microbiota’s ability to metabolize phenolic compounds and utilize these metabolites, thereby influencing the structure of microorganisms in the digestive tract [11]. In this review, we summarise the current state of knowledge, focusing on the impact of polyphenols on the development of gynaecological tumours in menopausal women, particularly endometrial cancer (EC), emphasising that polyphenol consumption leads to beneficial modifications in the structure of the gut microbiota.

## 2. Research Strategy Employed in the Review of Available Literature

**Figure 1:**
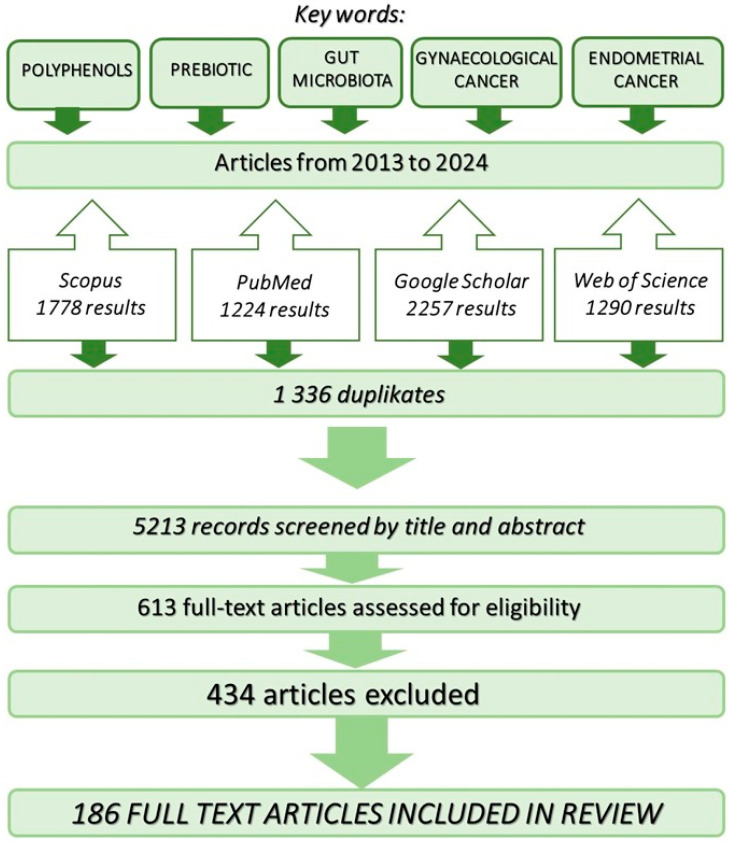
Research strategy employed in the review of available literature.

To analyse the data available in global literature, databases such as Scopus, PubMed, Web of Science, and Google Scholar were examined in December 2023. The databases were searched for both joint and separate instances of the keywords: “polyphenols”, “prebiotic”, “gut microbiota”, “gynaecological cancer”, and “endometrial cancer”, in Polish and English (Figure 1). After evaluating the titles and synopses, articles that did not meet substantive criteria were excluded. The remaining research and review papers were subjected to a more detailed analysis to identify the most relevant publications. The bibliographies of all the selected articles were also examined to discover additional potentially relevant texts. The search was restricted to studies published between 2013 and 2024. Finally, 3448 publications were examined, of which 186 were included. A protocol was developed according to the Preferred Reporting Items for Systematic Reviews and Meta-Analysis Protocols 2015 statement.

## 3. Pathogenesis of Endometrial Cancer

Endometrial cancer (EC) is one of the most frequently diagnosed malignancies in women worldwide. The incidence of this cancer is constantly increasing and is dominant among female patients [12]. The disease primarily affects postmenopausal women, with a mean age of 61. EC occurrence is concentrated in North America and Western Europe and is attributed to the increasing incidence of obesity and dietary habits. Endometrial cell activity is controlled by both progesterone and oestrogen [13]. EC constitutes almost 90% of uterine carcinomas, presenting two molecular varieties. Most cases are type I endometrioid differentiation cancer, where the dominant risk factors include exposure to endogenous and exogenous oestrogens, insulin resistance, and obesity. Recent studies have shown enhanced survival rates in obese women with endometrial cancer compared to non-obese women, which is linked to the absence of phosphatase and tensin homologue (PTEN). Additionally, women with a body mass index exceeding 30 kg/m^2^ who have complex atypical hyperplasia exhibit reduced expression of stathmin 1 (STMN1) and increased expression of Kirsten rat sarcoma viral oncogene homologue (KRAS) compared to their non-obese counterparts [14].

The diagnosis of the disease is favorable at an early stage owing to characteristic presentations, mainly postmenopausal bleeding. Consequently, the cure rate is high, and the prognosis is poor [15,16].

An important risk factor for type I EC is Lynch syndrome, which is diagnosed by assessing the MLH1 or MLH2 mismatch repair gene. Women with this condition have a lifetime risk of 40–60% for developing endometrial cancer and are much younger than the median age in the general population (48 years vs. 63 years) [17].

Increased serum levels of oestrogens may double the incidence of EC, particularly type I. Oestrogens have a mitogenic effect on endometrial cells by stimulating glandular and stromal tissue during the menstrual cycle [18].

Oestrogens are steroid hormones derived from the gradual reduction in cholesterol and play a crucial role not only in women’s reproductive processes but also in non-reproductive functions [19]. Circulating in the blood in free or protein-bound forms, oestrogens exert diverse biological effects [20]. They promote cell proliferation in the endometrial tissue during the proliferative phase of the menstrual cycle. However, when produced in excessive amounts (hyperestrogenism), they can lead to excessive cell proliferation in the endometrium [21,22]. Hyperestrogenism is a disorder that typically co-exists with oestrogen-dependent diseases. Two types of hyperestrogenism are distinguished: (1) relative imbalance due to the action of progesterone, which may contribute to abnormal endometrial growth and EC, and (2) chronic imbalance, which may lead to abnormal bleeding or endometrial overgrowth [23]. Insulin directly stimulates the activity of cytochrome P450c17, an enzyme responsible for converting progestogens into androgens; hence, hyperinsulinaemia may cause hyperandrogenism [24]. Five different androgens are produced in women: dehydroepiandrosterone sulphate (DHEAS), dehydroepiandrosterone (DHEA), androstenedione (A4), testosterone, and dihydrotestosterone (DHT). Testosterone and DHT are the most potent hormones, with DHT primarily produced from testosterone in the peripheral tissues, including the endometrium [19]. Obesity leads to the development of hyperandrogenaemia (increased levels of androgens from the adrenal glands, DHEA, and DHEAS). In turn, adipose tissue generates aromatase activity, which induces the conversion of androgens into oestrogens [25]. There is a correlation: the more adipose tissue there is, the higher the degree of androgen conversion to oestrogens. Aromatase is encoded by the CYP19A1 gene located on chromosome 15 in the 21.2 region of its long arm (15p21.2) in the endoplasmic reticulum of oestrogen-producing cells [25]. Aromatase expression depends primarily on three factors: (1) activation of tissue-specific promoters and transcription of the first exons associated with the promoter, (2) alternative splicing, and (3) the availability of various transcription factors [26].

Prolonged exposure to excess oestrogen increases the risk of developing type 1 EC [25]. Additionally, an elevated risk of EC has been observed with higher concentrations of parent oestrogens compared to their androgenic precursors [21]. Increased EC risk is noted in postmenopausal women despite a significant decrease in oestrogen levels during this period [21]. This is because, after menopause, some androgens are still produced by certain organs (adrenal glands, extragonadal subcutaneous fat tissue, liver, heart, and brain) and can be converted into oestrogens through aromatisation or into androgen metabolites [26]. However, it is unclear whether androgens influence the risk of oestrogen-dependent tumours by serving as oestrogen precursors or if alternative metabolic pathways of androgens may increase the risk [21]. Without a doubt, in studies conducted on women aged 50–79, it has been shown that an increased concentration of unconjugated oestradiol relative to testosterone increases the risk of EC, especially among women with a BMI below 30, and a high level of androstenedione increases the risk primarily among women with a BMI below 25 [21]. Oestrogen and its metabolites can induce the hyperproliferation and transformation of endometrial cells by increasing cell proliferation and DNA damage [25]. The prevalence of endometriosis, obesity, and the use of hormonal replacement therapy in women in developed countries is increasing, which justifies the assumption that EC may become more common in the future [23].

## 4. The Impact of Gut Microbiota on Carcinogenesis

The microbiota is associated with numerous pattern recognition receptors (PRRs), which serve as antibacterial mediators and promote cell death. The most prevalent PRRs are Toll-Like Receptors (TLRs), which are activated in several cell types, including macrophages, myofibroblasts, epithelial cells, and cancer cells [27]. The interaction between the microbiome, inflammation, and genes is believed to contribute to the development of cancer by inducing DNA damage and changes in metabolite pathways [28]. Various mechanisms through which bacteria can induce carcinogenesis have been proposed, including (1) promoting cell proliferation or death, (2) altering host cell metabolism, (3) disrupting the functioning of the immune system, and (4) causing DNA damage through the activation of reactive oxygen species (ROS) [20,27,28]. Generally, dysbiosis of the gut microbiota can induce tumourigenesis in other organs colonised by microorganisms, such as the skin, oral cavity, and reproductive system [29]. In this review, we summarise the current state of knowledge, focusing on the relationship between gut microbiota, inflammation, oestrogen metabolism, and dietary interventions aimed at stabilising oestrogen levels in the body.

## 5. The Impact of Gut Microbiota on Oestrogen Synthesis

The gut microbiota performs various crucial functions and plays a key role in maintaining homeostasis in the body. The gastrointestinal microbiome is primarily inhabited by five main types of bacteria: *Firmicutes*, *Bacteroidetes*, *Actinobacteria*, *Proteobacteria*, and *Fusobacteria* [2,28]. Dysbiosis, characterised by overgrowth of facultative anaerobic bacteria and a decrease in diversity and balance, can trigger an abnormal immune response, leading to reduced tolerance to commensal microbiota. It can also disrupt immune responses, causing inflammation, oxidative stress, and insulin resistance [2]. New evidence suggests that dysbiosis in the gastrointestinal and reproductive systems may play an active role in the progression and metastasis of gynaecological cancers such as cervical, endometrial, and ovarian cancers [30]. Gut dysbiosis should be considered a significant risk factor for oestrogen-dependent gynaecological cancers, mainly because some bacteria present in the intestines can metabolise oestrogens, collectively known as the estrobolome [30]. The estrobolome can modulate circulating oestrogen levels, which in turn modifies the structure of the vaginal microbiome, making the estrobolome indirectly involved in endometrial carcinogenesis [28]. Therefore, current research has focused on exploring methods to modulate the gut microbiome in the prophylaxis of oestrogen-dependent cancers through bariatric surgery, faecal microbiota transplantation, pharmaceutical methods (such as metformin), and nutraceutical approaches (such as genistein), which have shown beneficial results in treating the metabolic aspects of gynaecological cancers in several studies [31]. The structure of the estrobolome is likely influenced by factors such as age, ethnic origin, diet, exposure to xenobiotics (e.g., alcohol), and antibiotic use (e.g., ampicillin leads to an increase in conjugated oestrogens in faeces and decrease in oestrogen levels in women’s urine) [29].

Produced by the ovaries, oestradiol is the primary oestrogen in menstruating women, while during menopause, oestrone predominates and is produced in peripheral tissues from the steroid precursor androstenedione. Unlike oestriol, oestrone can be converted into oestradiol [32]. Oestrone, oestradiol, and oestriol are hydroxylated by oxidative enzymes of the cytochrome P450 family, resulting in the formation of 2-hydroxyestrogen (2-OH-E), 4-hydroxyestrogen (4-OH-E), and 16-hydroxyestrogen (16α-OH-E) [33]. These metabolites undergo further metabolism through methylation, glucuronidation, and sulfonation, leading to the formation of conjugated forms and changes in their structure and bioavailability [34]. Conjugation transforms oestrogens into less active, more polar, and water-soluble forms, facilitating their excretion through bile and urine [34]. Oestrogen glucuronides are the primary products of oestrogen metabolism during hepatic phase II [35]. They can be deconjugated in the intestines by bacteria that produce ß-glucuronidase, leading to the reabsorption/recycling of oestrogens into the bloodstream, enabling them to bind to oestrogen receptors. Activation of these receptors increases the number of cells in the G0/G1 phase of the cell cycle, promoting proliferation and influencing the tissues [35]. Most deconjugated oestrogens are reabsorbed, while the remaining are excreted from the body through bile, urine, and faeces [20]. Gut microorganisms also break down indigestible dietary polyphenols, resulting in the synthesis of oestrogen-like compounds [36]. Moreover, certain enzymes synthesised by gut microbes can reactivate oestrogens from their inactive glucuronides [37]. It is also possible that the estrobolome serves as a reservoir for oestrogens in the intestine and is capable of generating locally acting oestrogenic metabolites [37].

Β-Glucuronidase synthesised by certain bacterial strains was identified as a factor involved in oestrogen metabolism as early as 1944 [35]. In contrast, the estrobolome was first defined in 2011 [30]. The proposed mechanism involves the modulation of the gut-liver circulation and the reabsorption process of free oestrogens through the activation of oestrogen receptors [38]. For example, a reduction in the diversity of gut microbiota leads to a decreased rate of oestrogen metabolism due to the lack of metabolising bacteria, resulting in a decrease in circulating oestrogen levels and may contribute to the onset of obesity and metabolic syndrome. Conversely, an increased abundance of ß-glucuronidase-producing bacteria leads to elevated levels of circulating oestrogens, increasing the risk of diseases related to hyperestrogenism, including endometrial hyperplasia and cancer [39]. Elevated overall oestrogen levels can also induce changes in the functioning of the immune system, further influencing the structure of the gut microbiome [40]. Any imbalance in the gut microbiota affects the hormonal balance in a woman’s body.

It should be noted that elevated β-glucuronidase activity is observed within cancerous tissue; however, this is associated with necrosis of the tumour tissue, which releases the enzyme from cells, rather than with overexpression of this enzyme in tumour cells [41,42]. For some time, the possibility of modifying cancer cells to expose β-glucuronidase on their surface has been explored (such as gene-directed enzyme prodrug therapy GDEPT, antibody-directed enzyme prodrug therapy ADEPT, or ultrasound-directed enzyme prodrug therapy UDEPT). This could allow the utilisation of glucuronides of cytotoxic compounds as prodrugs because β-glucuronidase catalyses the hydrolysis of β-glucuronides into their active aglycones [42]. This is significant because of the poor pharmacokinetics and selectivity of low-molecular-weight anticancer drugs, contributing to their relatively low effectiveness [42]. In the case of smaller tumours unaffected by necrosis, glucuronide activation does not occur because of the lack of extracellular β-glucuronidase [41].

It has been observed that over 60 types of gut bacteria, including key types such as *Bacteroidetes*, *Firmicutes*, *Verrucomicrobia*, and *Proteobacteria*, as well as *Lactobacillus*, *Bifidobacterium*, *Enterococcus*, and others, synthesize β-glucuronidase [35]. Approximately 112 β-glucuronidase genes have been identified and grouped into six classes expressed in four bacterial phyla [43]. In addition to β-glucuronidase production, it is presumed that the gut flora may also influence oestrogen balance through other enzymatic pathways such as the β-glucosidase and sulfatase pathways [35,36]. Reduced β-glucuronidase activity in the intestines has been shown to be significantly correlated with lower counts of Lactobacillaceae and Streptococcaceae and increased counts of Ruminococcaceae [35]. Flores et al. [44] found that, in postmenopausal women, oestrogen levels were closely associated with the quantity and diversity of faecal microbiota, with the most correlated taxa being *Clostridia* and three genera from the Ruminococcaceae family. Active substances in food can regulate the structure of the gut microbiota. Among natural food components, compounds with prebiotic activity have a positive effect on the microbiota. This group includes indigestible oligosaccharides, whose positive influence on the structure of the gut microbiota and overall health has been proven in numerous studies [2,10,11]. However, recent studies have also shown that polyphenols, which are secondary metabolites produced by plants, exhibit prebiotic properties.

## 6. Prebiotics

Prebiotics are food components that have a positive impact on well-being and health by selectively stimulating the growth and/or activity of gut microflora. This, in turn, has a positive effect on the structure of the intestine and provides health benefits to the host [9,45]. The primary goal of supplementing the diet with prebiotics is to increase the population of beneficial bacteria, eliminate pathogens, and induce lasting changes in the composition of gut flora [5]. Mitigating gut dysbiosis is associated with reduced autoimmune responses, decreased inflammation, and improved intestinal integrity by enhancing protein expression in the intestinal epithelium [6].

### 6.1. Prebiotic Properties of Polyphenols

The presence of an increased number of gut bacteria producing β-glucuronidase and other enzymes involved in processes that promote deconjugation reactions of oestrogen metabolites may result in greater reabsorption of free oestrogens and an increased risk of oestrogen-dependent cancers [46]. This phenomenon is as perilous as a deficiency in β-glucuronidase, which can lead to metabolic issues that increase the risk of cancer, such as obesity [39]. β-Glucuronidase activity is modulated by diet and microbiota. For instance, a diet rich in fat or protein is associated with a high level of β-glucuronidase in faeces, whereas a fibre-based diet reduces the activity of this enzyme [36]. Some food components (phytoestrogens) can bind to oestrogen receptors and compete with endogenous oestrogens. This leads to a reduction in the pool of free oestrogens in the body, the excess of which increases the risk of oestrogen-dependent gynaecological cancers [25].

Higher levels of oestrogen in the body positively influence the diversity and composition of the gut microbiome, thereby reducing the availability of β-glucuronidase and increasing oestrogen excretion. Conversely, when endogenous oestrogen synthesis decreases, the gut microbiome structure shifts in favour of bacteria synthesising β-glucuronidase, leading to increased oestrogen reabsorption and the maintenance of systemic oestrogen homeostasis [47]. Therefore, it is essential during menopause to influence the gut microbiome structure to increase the abundance of bacteria that synthesise this enzyme. Maintaining a balance between various bacterial strains at different stages in a woman’s life is crucial. During the menopausal period, increased enzymatic activity of gut β-glucuronidase potentially plays a protective role in preventing diseases and improving health [47]. Studies have shown that β-glucuronidase is synthesised by over 60 types of Gram-negative gut bacteria, with key strains including *Bacteroidetes*, *Firmicutes*, *Verrucomicrobia*, *Proteobacteria*, *Lactobacillus*, *Bifidobacterium*, *Enterococcus*, *Citrobacter*, *Propionibacterium*, *Clostridium*, *Collinsella,* and *Streptococcus* [35,48].

The prebiotic properties of polyphenols, and thus their impact on the gut microbiota, stem from their produced metabolites and involve (1) influencing the growth and metabolism of bacteria, (2) disrupting the functions of cell membranes of pathogenic microorganisms, (3) regulating the expression of tight junction proteins, and (4) balancing the synthesis of pro-inflammatory and anti-inflammatory T lymphocytes [12,49]. This directly affects the microbiota structure by stimulating the growth of probiotic strains and inhibiting the growth of pathogenic bacteria, as demonstrated in numerous studies (Table 1). It also prevents damage to the gut barrier and helps to maintain proper functioning. Meanwhile, the activation of the Nrf2 (nuclear factor erythroid 2-related factor 2) signalling pathway through PI3K/Akt (phosphoinositide-3-kinase/Akt) protects IPEC-J2 (intestinal porcine enterocyte cell line) cells from oxidative stress, preventing damage to the gut barrier, as demonstrated in a study using resveratrol [50]. Most polyphenols hinder biofilm formation and/or inhibit the activity of certain bacterial enzymes. For example, flavonols (kaempferol and myricetin) hinder the activity of bacterial helicase in Staphylococcus, while simultaneously increasing the permeability of the cell membrane cytoplasm [51]. In contrast, resveratrol exhibits antibacterial activity against *Salmonella enterica*, *Enterococcus faecalis*, and *Escherichia coli* [52].

**Figure 2:**
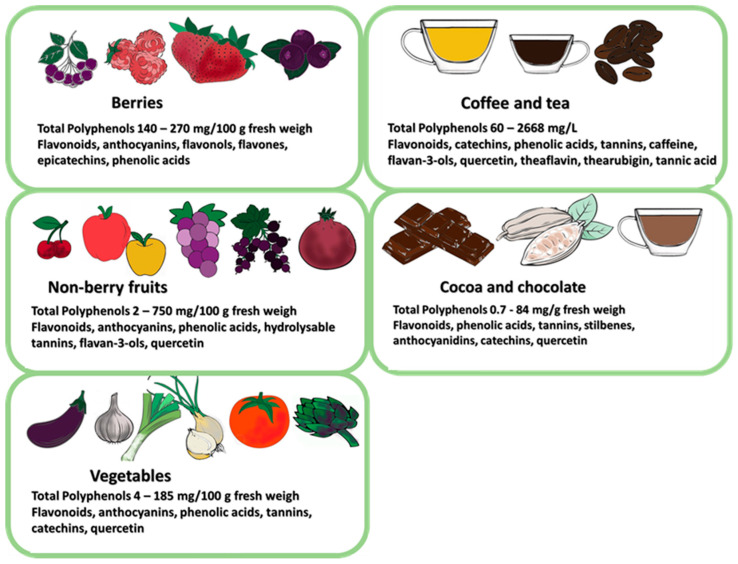
Food products with recognized health-promoting effects containing polyphenols.

The most significant advantage of polyphenols as health-promoting substances is their presence in food, which can be commonly and continuously consumed irrespective of an individual’s health status. Food products with recognised health-promoting effects, such as tea, coffee, cocoa, vegetables, and fruits, especially berries, meet these requirements (Figure 2).

#### 6.1.1. Prebiotic Action of Tea Polyphenols—A Review of Studies

Phenolic compounds present in tea (mainly EGCG, quercetin, theaflavin, thearubigin, and tannic acid) exhibit antioxidant properties [82]. The highest antioxidant activity, directly resulting from the highest relative polyphenol content, was observed in green and white tea [83,84]. Phenolic compounds in tea have selective bactericidal action. They damage bacterial cell membranes, thereby inhibiting the growth of various bacteria including *Bacillus cereus*, *Campylobacter jejuni*, *Clostridium perfringens*, *Escherichia coli*, *Helicobacter pylori*, *Legionella pneumophila*, and *Mycobacterium* spp. [85]. An example of selective bactericidal action is epigallocatechin gallate (EGCG), which can bind to peptidoglycans in the cell membranes of Gram-positive bacteria, causing their disruption, whereas Gram-negative bacteria are protected from such actions because of their outer membrane and negatively charged lipopolysaccharides, which repel catechins [85].

Interactions between tea polyphenols and the gut microbiota contribute to changes in the microbiota composition and the production of metabolites, including short-chain fatty acids (SCFAs), bile acids, and amino acids. The cleavage of glycosidic bonds in polyphenols results in glycan formation, which serves as a vital nutrient for the gut microbiota, especially *Bacteroidetes* [86]. In vitro studies have shown that tea polyphenols promote the growth of certain bacterial strains, including β-glucuronidase-synthesising *Bacteroides* and *Bifidobacterium* [87,88]. Polyphenols from green, black, and oolong tea stimulated the growth of *Bifidobacterium*, *Lactobacillus*, and *Enterococcus* in a study using human Caco-2 cells [89]. Flavonols may modulate the gut microbiota by influencing bacterial adhesion to intestinal cells, particularly *Lactobacillus acidophilus* LA-5 and *Lactobacillus plantarum* IFPL379 [90], while catechins stimulate the growth of the probiotic bacteria *Bifidobacterium* [86]. Kampferol present in tea leaves improves the integrity of the intestinal barrier and inhibits intestinal inflammation by reducing activation of the TLR4/NF-κB pathway (Toll-like receptors/Nuclear factor-kappaB). It also counteracts the dysbiosis associated with obesity, a risk factor for EC [82,91]. The gut microbiota of C57 mice showed an increase in the abundance of Bacteroidetes after administration of Ganpu tea (Pu-erh tea and mandarin peel) [92]. Similarly, Zheng et al. [93] obtained favourable results with this tea in studies conducted in rats, where an increase in the abundance of *Bifidobacterium*, *Lactobacillus*, and *Lactococcus* bacteria was observed in the gut microbiota. Liupao dark tea, used in traditional Chinese medicine as an anti-diabetic agent, administered to streptozotocin-induced diabetic rats, led to an increase in the abundance of *Bacteroides* [94].

#### 6.1.2. Prebiotic Effects of Coffee Polyphenols—A Review of Studies

Coffee is a rich source of polyphenols with strong antioxidant and health-promoting properties [95]. The main phenolic compounds found in coffee beans are phenolic acids (primarily gallic, chlorogenic, caffeic, and salicylic acids) and flavonoids (especially catechin, quercetin, and kaempferol) [96]. Regular consumption of coffee induces significant changes in the gut microbiota population. In the human faecal microbiota, coffee beans were found to increase the abundance of *Bifidobacterium*, *Bacteroides*, *Clostridium coccoides*, and *Eubacterium rectale*, while having no impact on the counts of *Lactobacillus* and *Enterococcus* [97]. Quercetin and its glycosides, which are present not only in coffee but also in tea and fruits, increased the abundance of *Akkermansia*, *Bacteroides*, *Parabacteroides*, and *Ruminococcus* in rats fed a high-fat sucrose diet [98]. Wistar rats orally administered soluble caffeinated or decaffeinated coffee solution showed an increase in the abundance of *Firmicutes* and *Bacteroidetes* compared to the control group [99]. Similarly, in Wistar rats with diet-induced metabolic syndrome, coffee pulp application led to a decrease in the *Firmicutes/Bacteroidetes* ratio and an increase in gut microbiota diversity [100,101]. These changes resulted in reduced obesity and improved glucose tolerance and systolic blood pressure. Studies on the long-term consumption of coffee by humans have revealed a higher abundance of *Bacteroides*, *Prevotella*, and *Porphyromonas* in the gut microbiota of individuals who consume more coffee. These individuals also exhibit lower levels of lipoperoxidation [102]. Chlorogenic acid is the predominant phenolic acid found in coffee. Depending on the type of coffee and its concentration in the brew, a cup of coffee can contain 27–121 mg of chlorogenic acid [103]. Administering C57BL/6 mice on an HFD with chlorogenic acids resulted in a reduction in the relative mass of subcutaneous and visceral adipose tissue (a risk factor for EC), improved the integrity of the intestinal barrier, and aided in the prevention of glucose metabolism disorders and metabolic endotoxaemia (a consequence of intestinal mucosal barrier damage) [104]. This study also observed an increase in the abundance of bacteria synthesising SCFA (*Dubosiella*, *Romboutsia*, *Mucispirillum*, and *Faecalibaculum*) and *Akkermansia*. Moreover, the application of chlorogenic acid led to a gut microbiota composition in HFD-fed mice similar to that in mice fed a standard diet (reducing the number of *Firmicutes* and increasing the number of *Bacteroidetes* and *Verrucomicrobia*). In other studies, chlorogenic acid reversed gut microbiota dysbiosis in HFD-fed mice by inhibiting the growth of *Desulfovibrionaceae*, *Ruminococcaceae*, *Lachnospiraceae*, and *Erysipelotrichaceae* and increasing the growth of *Bacteroidaceae* and *Lactobacillaceae* [105].

#### 6.1.3. Prebiotic Effects of Cocoa Polyphenols—A Review of Studies

The total polyphenol content in cocoa beans ranges from 40 to approximately 84 mg per 1 g, while chocolate contains approximately ten times fewer polyphenols [106]. Cocoa and its derivatives are a rich source of polyphenols, primarily flavanols, flavones, flavanones, flavonols, anthocyanidins, and phenolic acids, endowing them with anti-inflammatory and antioxidant properties [107,108]. These compounds are poorly absorbed in the small intestine; most reach the large intestine, where they are metabolised by the microbiota [109]. *Escherichia coli*, *Bifidobacterium*, *Lactobacillus*, *Bacteroides*, and *Eubacterium* play significant roles in the metabolism of cocoa polyphenols in the gastrointestinal tract [110]. Studies have demonstrated that consuming cocoa and dark chocolate leads to an increase in beneficial gut bacteria, such as *Lactobacillus* and *Bifidobacterium*, while inhibiting the growth of pathogenic bacteria, such as Clostridium perfringens [107,109,111]. Feeding rats a cocoa-containing diet resulted in a reduction in the abundance of *Bacteroides*, *Clostridium*, and *Staphylococcus* in faeces, positively modulating the gut immune system [112]. Similarly, positive prebiotic effects were observed in studies conducted on a pig model using flavonoid-enriched cocoa (increasing the abundance of *Lactobacillus* and *Bifidobacterium* and modulating the intestinal immune markers Tumour Necrosis Factor-α TNF-α and Toll-Like Receptors TLRs) [113].

#### 6.1.4. Prebiotic Effects of Fruit Polyphenols—A Review of Studies

Berry fruits and some non-berry fruits, such as blackcurrants, grapes, cherries, and apples, exhibit the highest phenolic compound content [52,114,115,116]. Ellagic acid and anthocyanins found in raspberries increased the abundance of *Lactobacillus* and *Akkermansia*, while decreasing the levels of *Bacteroides* and *Ruminococcus*, as demonstrated in an in vitro study [117]. Berries rich in phenolic compounds, such as phenolic acids, flavonols, and anthocyanins, contribute to the increased abundance of *Bifidobacterium*, *Lactobacillus*, *Akkermansia*, *Bacteroides*, and *Eubacterium* while decreasing the counts of *Pseudomonas*, *Salmonella*, *Staphylococcus*, and *Bacillus* [12]. Consuming berries alleviates the symptoms of intestinal inflammation by modulating pro-inflammatory cytokines and acts chemopreventively in colorectal cancer by regulating apoptosis, cell proliferation, and angiogenesis. In healthy individuals, berry consumption leads to an increase in *Bifidobacterium*, *Lactobacillus*, and *Akkermansia* levels [118,119]. In studies on mice fed a high-fat diet (HFD), an increase in the abundance of *Bifidobacterium*, *Desulfovibrio*, *Adlercreutzia*, *Helicobacter*, and *Flexispira* and a decrease in *Prevotella* were observed after the administration of berry extract [56]. Applying peach peel extract polyphenols to female mice on an HFD inhibited lipid accumulation and modified the gut microbiota composition, elevating the levels of *Lactobacillus*, *Bacteroides*, *Lachnospiraceae*, *Prevotellaceae*, *Alloprevotella*, *Akkermansia*, *Roseburia*, and *Ruminococcus* compared to the group not receiving polyphenols [120]. In vitro studies on phenolic compounds in grape seeds have shown an increased abundance of Bifidobacterium spp. and *Lactobacillus-Enterococcus*, while decreasing *Clostridium histolyticum* and *Bacteroides-Prevotella* counts [121]. Polyphenols from grapes and red wine increased the abundance of *Enterococcus*, *Prevotella*, *Bacteroides*, *Bifidobacterium*, *Bacteroides uniformis*, *Eggerthella lenta*, and *Blautia coccoides-Eubacterium rectale* and decreased the abundance of *Actinobacteria* and *Clostridium* spp. [122]. Anthocyanins, flavonoids, and neochlorogenic acids present in cherries increase the abundance of *Bacteroidetes*, *Firmicutes*, and *Proteobacteria* in a study using a Simulator of the Human Intestinal Microbial Ecosystem [123]. Studies on mice fed an HFD receiving phenolic acids, flavonoids, and anthocyanins from cranberries showed a decrease in the *Firmicutes* to *Bacteroidetes* ratio [124]. Similar results were obtained for red pitaya betacyanins in HFD-fed mice [125]. Pomegranate polyphenols increase the abundance of *Bacteroidetes* and reduce the levels of *Firmicutes* and *Proteobacteria* in the gut microbiota of mice [126]. Consuming apples with increased anthocyanin content leads to changes in the expression of immune cell genes and positively modifies the composition of faecal microbiota in healthy adults (decreasing *Streptococcus*, *Ruminococcus*, *Blautia*, and *Roseburia*, and increasing *Sutterella*, *Butyricicoccus*, and *Lactobacillus*) [127]. Similar results were achieved by Espley et al. [128], who found that feeding mice with a transgenic variant of “Royal Gala” apples enriched with flavonoids beneficially modulated the colon microbiota and reduced inflammatory biomarkers. A dried mix of fruits (apple, pear, watermelon, winter jujube, grape) and vegetables (Chinese cabbage, tomato, cucumber, carrot, cauliflower) given to C57BL/6 mice with induced metabolic syndrome resulted in modifications to the gut microbiota structure, decreasing the abundance of *Firmicutes*, *Syntrophomonadales*, and *Pseudomonadales* [129].

#### 6.1.5. Prebiotic Effects of Vegetable Polyphenols—A Review of Studies

The overall content of polyphenols in vegetables ranges from 4 to 185 mg/100 g of fresh weight, with flavonoids, anthocyanins, phenolic acids, tannins, catechins, and quercetin being the most prominent [114,130]. In vitro studies have shown that artichokes exhibit potential prebiotic effects [131,132]. A medium containing artichoke extract led to an increase in beneficial lactic acid bacteria and *Bifidobacterium* compared to that observed with inulin and sucrose, directly translating to higher levels of short-chain fatty acids (SCFA). Elevated SCFA concentrations reduce the pH in the intestines, inhibiting the growth of Enterobacteriaceae, which produce lipopolysaccharides [129]. It is noteworthy that artichokes contain the oligosaccharide inulin [132], which is known for its prebiotic properties. However, the fact that artichoke extract performed more effectively than inulin alone confirms that other components of artichokes, mainly phenolic compounds (especially phenolic acids, flavonoids, and sesquiterpene lactones), also exhibit prebiotic properties. In an in vitro study, tomato powder positively influenced the growth of Bifidobacterium longum and Bacteroides and promoted the growth of *Lactobacillus casei* [133]. Fermented tomatoes administered to obese mice modify the composition of the gut microbiota [134]. This product inhibits the growth of bacteria associated with obesity (such as *Clostridium*, *Olsenella*, and *Mucispirillum*) and promotes the beneficial growth of bacteria negatively correlated with body mass, such as *Roseburia*, *Coprococcus*, and *Oscillospira*. Studies using piglets as a physiological model of human metabolism found that after only 14 days of tomato powder supplementation, there was an increase in the *Bacteroidetes* and *Firmicutes* ratios [135]. Daily consumption of 200 g of cooked broccoli and 20 g of raw radish by adults reduced the relative abundance of Firmicutes and increased the relative abundance of *Bacteroidetes* and *Bacteroides* compared to the control group [136]. Polyphenols from carrots may enhance the growth of *Lactobacillus rhamnosus* and *Bacteroides*, while reducing *Clostridiales*, *Ruminococcus*, *Coprococcus*, and *Oscillospira*. Consuming whole carrots is more beneficial than using an extract [12,137]. Carrot fibre containing associated polyphenols increased the *Firmicute/Bacteroidetes* ratio and SCFA synthesis in both in vivo (mice) and in vitro (human faeces) studies [137,138]. Flavonoids from parsley, when included in the diet of mice for 6 weeks, increased SCFA synthesis in the intestine, correlating with the growth of bacteria synthesising SCFA [66].

#### 6.1.6. Metabolism of Phenolic Compounds by Estrobolome

**Figure 3:**
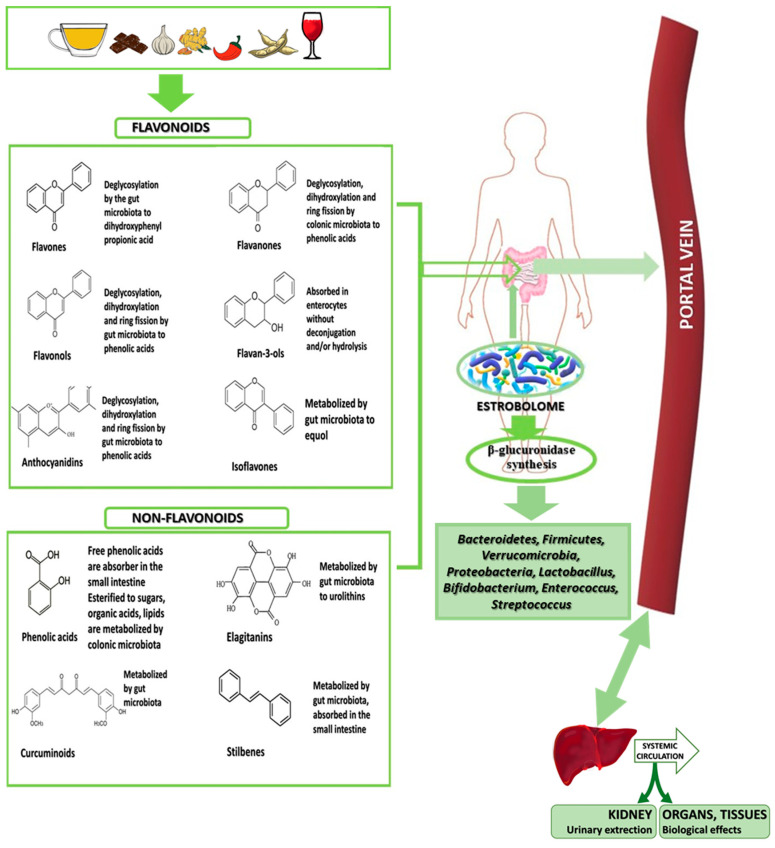
Metabolism of polyphenols.

Polyphenols are present in plants as glycosides and complex oligomeric structures. Polyphenols exhibit low bioavailability in the small intestine [87], making the involvement of microbiota crucial for their absorption and, consequently, for human health. Certain bacteria are associated with polyphenol metabolism. Two main pathways through which the microbiome influences the hormonal balance of the reproductive system have been identified: (1) in the deconjugation-independent pathway, some phytoestrogens present in food are metabolised by specific gut bacteria into bioactive compounds; and (2) in the deconjugation-dependent pathway, oestrogens excreted by the liver into the intestinal lumen can be deconjugated by hydrolytic enzymes (β-glucuronidases and β-glucosidases), potentially increasing their reabsorption through enterohepatic circulation [48]. The most frequently mentioned include *Flavonifractor plautii*, *Slackia equolifaciens*, *Slackia isoflavoniconvertens*, *Adlercreutzia equolifaciens*, *Eubacterium ramulus*, *Eggerthella lenta*, *Lactobacillus*, and *Bifidobacterium* spp., with different bacteria showing affinity for different groups of polyphenols [52]. However, *Firmicutes* and *Bacteroidetes* are the main colonic bacteria responsible for the metabolism of polyphenols [139]. Polyphenol metabolism occurs via the involvement of bacteria in the intestinal lumen (Figure 3). Once polyphenols reach the distal part of the intestine, they undergo hydrolysis and metabolism by intestinal enzymes and gut microbiota. Subsequently, the transformed polyphenols reach the liver through the portal circulation, where numerous metabolites are produced through two different processes. The next step in metabolism involves absorption, with the participation of glucuronidation, sulfation, and catechol transferases, resulting in the formation of sulphate, glucuronide, and methyl conjugates [12]. The bioavailability of polyphenols depends on factors such as sex, transit time in the intestine, and interactions with the gut microbiome. Gut microbiota can transform dietary lignans and produce phytoestrogens, namely enterodiol and enterolactone [12]. The phytoestrogenic properties of phenolic compounds exemplify their prebiotic effect.

## 7. The Microbiota Structure and Diet-Related Factors in Oestrogen-Dependent Gynaecological Cancers

Diet-dependent factors influencing the development of oestrogen-dependent gynaecological cancers, including EC, include obesity and associated metabolic diseases, such as type 2 diabetes, insulin resistance, and metabolic syndrome [82]. A physiological marker of such cancers, as well as coexisting diet-related diseases, is disturbances in the efficiency of the antioxidant system and a progressing state of inflammation—factors whose connection to the structure of the gut microbiome has been demonstrated [2,52]. The gut microbiota can regulate the development of metabolic disorders by modulating appetite, energy acquisition, and absorption; regulating the function of the intestinal barrier; controlling chronic inflammation; regulating lipid and glucose metabolism; and ultimately influencing weight gain and fat storage in the liver and adipose tissue [52]. Gut flora also participates in appetite regulation by modifying gut hormones secreted by enteroendocrine cells, such as ghrelin, peptide YY, leptin, and GLP-1 [137]. The relationship between excessive body weight and the structure of the gut microbiota has been demonstrated (Table 2).

Many studies conducted in animals and humans have shown that obesity may be associated with the prevalence of two dominant types of bacteria: *Firmicutes* and *Bacteroidetes* [87,152]. It is most commonly observed that in obese individuals, Firmicutes bacteria are more abundant than *Bacteroidetes*, which is associated with higher energy absorption from food and a low-grade inflammatory state [52]. A high-fat and/or high-carbohydrate diet stimulates the growth of *Firmicutes*, *Prevotella*, and *Methanobrevibacter*, at the expense of beneficial *Bacteroides*, *Bifidobacterium*, *Lactobacillus*, and *Akkermansia* [153]. This results in reduced short-chain fatty acid (SCFA) synthesis, negatively affecting the integrity of the intestinal mucosa and causing chronic inflammation. Impairment of the intestinal barrier leads to the entry of bacterial metabolites into the circulatory system, which may disrupt insulin sensitivity, glucose metabolism, and immune homeostasis [154]. It should be noted, however, that some authors have not demonstrated a correlation between the *Firmicutes/Bacteroidetes* ratio and obesity, or they have observed that in obese individuals, the *Firmicutes/Bacteroidetes* ratio is lower than in lean individuals [155], but these are isolated reports.

In individuals with type 2 diabetes, similar to those with obesity, an elevated *Firmicutes/Bacteroidetes* ratio is observed [156], which can be explained by the fact that excess body weight is a significant risk factor for this type of diabetes. Individuals with type 2 diabetes also exhibit a decreased abundance of probiotic strains such as *Lactobacillus*, *Bacteroides fragilis*, and *Faecalibacterium prausnitzii* [157,158]. In addition to *Bacteroidetes* and *Firmicutes*, type 2 diabetes is associated with bacteria such as *Clostridium leptum*, *Faecalibacterium prausnitzii*, *Bacteroides*, *Bifidobacterium*, *Akkermansia muciniphila*, and *Escherichia coli* [159]. It has also been noted that butyrate-producing bacteria (*Faecalibacterium prausnitzii*, *Clostridium leptum*, *Eubacterium rectale*, *Roseburia*) are present in lower quantities in individuals with type 2 diabetes compared to healthy individuals [160]. However, some studies have questioned the validity of using the *Firmicutes/Bacteroidetes* ratio to assess dysbiosis in individuals with diabetes. Even though a reduced *Firmicutes/Bacteroidetes* ratio has been described in individuals with obesity and metabolic syndrome, its increase has been positively correlated with reduced glucose tolerance [161]. It is also worth noting that a positive association between *Lactobacillus* and type 2 diabetes has been observed, although some *Lactobacillus* species exhibit anti-inflammatory properties: they induce the production of anti-inflammatory IL-10, which improves muscle insulin sensitivity [160]. Conflicting results obtained by different authors may be the result of the use of various types of antidiabetic therapies in the patients studied.

Insulin resistance is the fundamental pathophysiology underlying type 2 diabetes and metabolic syndrome [82]. Insulin resistance is associated with an elevated *Firmicutes/Bacteroidetes* ratio, which is similar to obesity and type 2 diabetes [162]. The relative abundance of *Firmicutes* is positively correlated with insulin sensitivity [163]. Insulin resistance may result from chronic inflammation, during which pro-inflammatory factors (TNF-α, IL-6, and others) can phosphorylate the insulin receptor substrate, converting it into serine, negatively impacting insulin signalling [164]. It has been shown that *Bacteroidetes* and *Bifidobacterium* contribute to improving mucosal barrier function, resulting in reduced inflammation, improved glucose tolerance, and decreased oxidative stress [165]. For example, *Bacteroides vulgatus* and *Bacteroides dorei* maintain intestinal wall integrity by regulating their expression and reducing lipopolysaccharide production [161]. Lipopolysaccharides are essential for the life processes of Gram-negative bacteria, but on the other hand, they are also one of the factors contributing to their pathogenicity, inducing an inflammatory reaction in the body. Dysbiosis contributes to increased gut permeability, and consequently, the translocation of bacteria and bacterial lipopolysaccharides into the bloodstream. Metabolic endotoxaemia is considered a key factor involved in the pathogenesis of insulin resistance and type 2 diabetes [161].

## 8. The Effect of Polyphenols on Endometrial Cancer

Similar to other cancer diseases, oesophageal cancer (EC) is characterised by the presence of chronic oxidative stress, which triggers inflammation and results from intense metabolism due to continuous cell proliferation and mutations in mitochondrial DNA [166]. Oxidative stress not only leads to the occurrence of inflammatory reactions but also activates the transcription of genes for various inflammatory factors, depending on proteins from the nuclear factor κB (NF-κB) family [167]. Strong oxidative stress can play a significant role in carcinogenesis, leading to damage and mutations in tumour suppressor genes, and chemical modification of genetic material by ROS constitutes the first stage in the process of mutagenesis and carcinogenesis [168]. Owing to the variable redox status of cancer cells, they are susceptible to therapies involving ROS manipulation.

Although phenolic compounds are commonly consumed, their health-promoting properties have only recently been recognised. They are gaining increasing attention owing to their strong antioxidant properties and significant impact on preventing the development of various diseases associated with oxidative stress, such as cancer. In a study conducted on 410 women with EC and 395 healthy women, it was found that total phenol intake may reduce the risk of EC occurrence [169], which could enhance the overall antioxidant protection of the body. The antioxidant properties of phenolic compounds may result from several mechanisms, including (1) neutralisation of free radicals, (2) inhibition of ROS formation by inhibiting certain enzymes or chelating trace metals involved in free radical production, and (3) enhancement of antioxidant defences [170,171].

Several mechanisms of the anticancer activity of phenolic compounds have been proposed, including both antioxidant and pro-oxidant activities, as well as interference with cellular functions, including (1) inhibition of phase I enzyme synthesis and induction of phase II enzymes, (2) stimulation of DNA repair, (3) cell cycle arrest and apoptosis induction, and (4) inhibition of cancer cell proliferation [82,171]. Phenolic compounds contain hydroxyl groups capable of donating a hydrogen atom or electron, and an extended conjugated aromatic system, making them chemically ideal for neutralising ROS [170]. Additionally, their action involves activating intracellular pathways, such as the arachidonic acid-dependent pathway, nuclear factor kappa B (NF-κB), mitogen-activated protein kinase (MAPK) pathway, and phosphatidylinositol 3-kinase/protein kinase B (PI3K/Akt) pathway, as well as stimulating epigenetic modulations that regulate the body’s immune response [172].

## 9. Plant-Based Diets Show Anti-Cancer Effects through Their Polyphenol Content

Therefore, dietary patterns based on plant products are of great interest. Numerous epidemiological studies have shown an association between plant-based diets and reduced risk of cancer [173]. Current evidence suggests that adherence to the Mediterranean and vegetarian diets is associated with numerous health benefits. This is due to the presence of extra virgin olive oil, nuts, herbs, red wine, vegetables, fruits (unprocessed or minimally processed), legumes, whole grains, and beverages, such as tea, coffee, and red wine [174,175]. All of these products are excellent sources of polyphenols, including isoflavones (Figure 2), which makes the Mediterranean diet exhibit strong antioxidant and anti-inflammatory properties. As a result, it counteracts DNA damage, cancer cell proliferation, angiogenesis, and inflammation and protects cell membranes from metastases, making it an effective and straightforward method of dietary therapy in cancer prevention and treatment [176]. Studies conducted in Greece have shown that individuals adhering to the Mediterranean diet consume between 1000 and 2000 mg of polyphenols daily, Spaniards consume approximately 700–3000 mg of polyphenols, and French adults consume an average of 1193 ± 510 mg of polyphenols per day [177]. The Mediterranean diet is often compared to other healthy dietary patterns such as the Nordic diet, Dietary Approaches to Stop Hypertension (DASH), or vegetarian diet [176].

An increasingly observed dietary pattern is the so-called oriental diet, which is commonly practised in Asian countries [178]. The basis of this dietary pattern includes plant products, primarily soy, which is a rich source of isoflavones. Isoflavones, as phytoestrogens, can bind to oestrogen receptors, leading to a reduction in free oestrogen levels in the body and lowering the risk of oestrogen-related gynaecological cancers [25]. Isoflavones bind to oestrogen receptors differently from oestrogens, acting as tumour suppressors [179]. It has been shown that in Asian populations, which consume significantly more soy products than Western populations, hormone-dependent cancers, including EC, are less common [179]. It has also been demonstrated that among Asian immigrants living in Western countries who consume a Western diet, the risk of hormone-dependent cancers is similar to that in Western populations [180]. The most important isoflavone present in soy is genistein, which shares structural similarity with oestradiol and has proven anti-proliferative effects on cancer cells, including EC [181,182].

## 10. Supplements or Food—Dietary Prevention of Oestrogen-Dependent Gynaecological Cancers

Supplements provide a known, appropriate active dose of the active ingredient, which is an undeniable advantage. Additionally, active substances present in dietary supplements may have modified bioavailability and may also lead consumers to choose them over a healthily balanced diet that provides compounds that modulate the gut microbiome. There might be a presumption of higher effectiveness of foods containing polyphenols compared to individual phenolic compounds due to the cumulative action of active substances present in the food, including phenolic compounds.

Plant-based foods contain not only polyphenols, but also numerous other phytonutrients (fibre, vitamins, vitamins, and minerals) and essential nutrients. In Poland, the average daily intake of polyphenols from food is 1756.5 ± 695.8 mg. They are primarily flavonoids (897 mg/day) and phenolic acids (800 mg/day) [183]. The main sources of polyphenols in the diet of the surveyed individuals (*n* = 10,728 adults, aged 45–69 years) were coffee, tea, and chocolate, with a smaller contribution from fruit and vegetables. In the Czech Republic, the average daily intake of polyphenols from food is 426 mg, primarily including chlorogenic acid (from potatoes, coffee, and plums), apigenin flavone, heneicosylresorcinol, ferulic acid (from wheat), and anthocyanin malvidin (from red wine) [184]. Similarly, in Germany, the daily polyphenol intake is less than 400 mg, with the primary sources being pome fruits and black tea [185]. Mediterranean and plant-based diets are rich in polyphenols because they are based on fresh plant products. For example, adherence to the Mediterranean diet leads to daily polyphenol intake ranging from 2590 to 3016 mg/day [186]. Because of the presence of other plant bioactive substances, such as essential unsaturated fatty acids, fibre, vitamins, provitamins, and mineral components, they are recommended as dietary therapies for obesity, type 2 diabetes, insulin resistance, and metabolic syndrome [186], which are risk factors for oestrogen-dependent gynaecological cancers, especially EC.

A good solution is the continuous education of society regarding the daily consumption of plant-based foods to increase the intake of phenolic compounds in the diet. Although dietary supplements are effective, their action is limited by the relatively low diversity of active substances, unlike unprocessed plant-based foods.

## 11. Summary and Perspectives

Research on gut microbiota and its significance in oestrogen-dependent gynaecological cancers continues to evolve. These results clearly indicate that the importance of gut microbiota in the treatment process of EC should not be underestimated. Profiling of the gut microbiome can be conducted to assess the levels of microorganisms encoding β-glucuronidase, which impacts oestrogen metabolism. Furthermore, regulating the gut flora using biologically active substances in individuals with gynaecological cancers may serve as a complementary treatment method alongside surgery, chemotherapy, or radiotherapy. Modulating the microbiome through dietary interventions can be beneficial in improving responses to cancer treatment and the overall quality of life. Understanding the correlation between polyphenols and the gut microorganisms that metabolise them is crucial for understanding their health benefits. Individual patient characteristics should not be overlooked, as they may influence the gut microbiota response to polyphenols and various bacterial metabolites. For example, in studies using polyphenol-rich tart cherries, a decrease in the abundance of *Bacteroides* and *Bifidobacterium* and an increase in the abundance of *Lachnospiraceae*, *Ruminococcus*, and *Collinsella* (which have the ability to metabolise polyphenols) were observed after five days in individuals with a high *Bacteroides* count. Conversely, individuals with a low *Bacteroides* count showed an increase in *Bacteroides*, *Prevotella*, and *Bifidobacterium* along with a decrease in the abundance of *Lachnospiraceae*, *Ruminococcus*, and *Collinsella* [123].

## Figures and Tables

**Table 1:** Influence of polyphenol-rich extracts and individual polyphenols on the composition of gut microbiota.

	Animal Model	Time	Dosage (mg/kg b.w.) (Body Weight)	Effects	Ref.
Apple polyphenol extract (49.27% epigallocatechin gallate, 10.09% epicatechin gallate, 15.49% epigallocatechin, and 7.18% epicatechin)	Male mice	12 week	125 and 500 mg/kg	Regulation of gut microbiota and its composition ↑ *Akkermansia* *↓ Lactobacillus*	[53]
Grape extract	Obese mice	11 weeks	1% *w*/*w* extract dissolved in water	Restoration of gut microbiota dysbiosis ↑ *Bifidobacteria*, *Akkermansia*, *Clostridiagenera* and *Firmicutes*/*Bacteroides*	[54]
Green pea (*Pisum sativum* L.) hull extract (quercetin and its derivatives (2836.57 μg of quercetin/g of extracts), kaempferol trihexoside (1482.00 μg of quercetin/g of extracts), and catechin and its derivatives (1339.91 μg of quercetin/g of extracts))	Mice	7 days	100 mg/kg	Regulation of gut microbiota ↑ *Firmicutes/Bacteroidetes* and promoting the growth of *Lactobacilaceae* and *Lachnospiraceae*	[55]
Blueberry polyphenols extract	Obese mice	12 weeks	200 mg/kg	Alteration of gut microbiota composition and modulation of gut microbiota composition ↑ *Proteobacteria*, *Deferribacteres Bifidobacterium*, *Desulfovibrio*, *Adlercreutzia*, *Helicobacter*, *Flexispira* ↓ *Actinobacteria*, *Flexispira*, *Adlercreutzia*, *Prevotella*	[56]
Blueberry anthocyanin extract	Obese mice	12 weeks	200 mg/kg	Modulation of gut microbiota, Improvement of insulin sensitivity	[57]
Honey polyphenols (caffeic acid, chlorogenic acid, rutin, etc.)	Adult male rats	7 days	10.5 mg/kg	Enhancement of resistance to oxidative stress through modulation of gut microbiota ↓ *Bacteroides*, *Corynebacterium*, *Proteus*	[58]
Aronia melanocarpa extract	Rats	4 weeks	200 mg/kg	Reinforcement of the gut barrier ↑ *Lactobacillus*, *Enterobacteriaceae*, *Enterobacteriaceae*, *↓ Lachnospiraceae*, *Phascolarctobacterium*, *Clostridiales*	[59]
Grape seed proanthocyanidin	Male mice	20 days	250 mg/kg	Regulation of gut microbiota	[60]
Grape seed proanthocyanidin extract ((monomeric (21.3%), dimeric (17.4%), trimeric (16.3%), tetrameric (13.3%), and oligomeric (31.7%) proanthocyanidins)	Rats	8 days	500 mg/kg	Effectiveness in modifying microbiota ↑ *Bacteroidetes*, ↓ *Firmicutes*, Modifications in the microbiota led to changes in the profile of short-chain fatty acids,	[61]
Canarium album extract	Male mice	4 weeks	10 mg/kg	↑ *Firmicutes*, *Verrucomicrobia*, ↓ *Bacteroidetes*	[62]
Arecanut (*Areca catechu* L.) seed polyphenols (28,284.5896 μg/g catechin, 5628.2237 μg/g procyanidin B1, 5486.037 μg/g quinic acid, 1150.3457 μg/g proanthocyanidin B2, etc.)	Female rats	90 days.	400 mg/kg.	↓ *Alistipes* ↑ *Phascolarctobacterium*, *Blautia*, *Faecalibaculum*, *Fusicatenibacter*, *Ruminococcaceae*, *Allobaculum*, *Negativibacillus*, *Turicibacter*, *Lactobacillus*	[63]
Polyphenol extract and essential oil (TKO) from Amomum tsao-ko Crevost et Lemaire (tsao-ko) (50.00% epicatechin, 30.60% kaempferol, 7.10% protocatechuic acid, 2.58% vanillic acid, etc.)	Male hamsters	6 weeks.	1000 mg/kg	↑ *Bacteroidetes*	[64]
Green tea extract (49.27% epi-gallocatechin gallate, 10.09% epicatechin gallate, 15.49% epigallocatechin, and 7.18% epicatechin)	Male mice	28 days	360 mg/kg	↑ *Lactobacillus*, *Akkermansia*, *Blautia*, *Roseburia*, *Eubacterium*, Regulation of gut microbiota dysbiosis induced by antibiotics	[65]
Parsley (*Petroselinum crispum*) flavonoids extract (apigenin-7-*O*-glucuronide, diosmetin-7-*O*-glucoside, kaempferol-7-*O*-glucoside, and scutellarin)	Mice	6 weeks	50 mg/kg	Stimulation of probiotics and microbiota producing short-chain fatty acids	[66]
*Lycium ruthenicum* anthocyanins (petunidin (95.17%))	Mice	12 weeks	50, 100, 200 mg/kg	Alleviation of colonic barrier dysfunction	[67]
Polyphenols					
Curcumin	Mice	7 days	50 or 150 mg kg	Strengthening the intestinal barrier, Modulating the abundance of bacteria: ↑ Odoribacter/*Coprococcus*, *↓ Turicibacter*, *Enterococcaceae*	[68]
Baicalin	Male mice	7 days	100 mg/kg	Restoration of the normal microbiological composition of the intestines, ↑ *Halomonas smyrnensis*, *Citromicrobium* sp. *WPS32*, *Eubacterium* sp. *CAG 86*, ↓ *Parabacteroides johnsonii*, *Bacteroides*,	[69]
Caffeic acid	Male mice	12 weeks	50 mg/kg	Regulation of gut microbiota, ↑ *Muribaculaceae* ↓ *Lachnospiraceae*	[70]
Epigallocatechin- 3-gallate	Male mice	2 weeks	50 mg/kg	Significant improvement in gut microbiota dysbiosis caused by the methionine—choline-deficient diet, ↑ *Bacteroidetes*, *Alloprevotella*, *Bifidobacteria*, *Lactobacillus* ↓ *Firmicutes*	[71]
Ferulic acid	Rats	8 weeks	30 mg/kg	↓ *Lactobacillus*, *Ruminococcus*, *Oscillibacter*, *Desulfovibrio*, ↑ *Akkermansia*, *Phascolarctobacterium*, *Turicibacter*	[72]
Genistein	Mice	8 weeks	40 mg/kg	↓ *Firmicutes/Bacteroidetes*, ↓ *Proteobacteria*, *Ruminococcus*, *Helicobacter* ↑ *Bacteroides*, *Prevotella*	[73]
Kaempferol	Mice	6 weeks	100 mg/kg	↑ *Actinobacteria*, *Verrucomicrobia*, *Akkermansia*, *Alloprevotella*, *Bacteroides*, *Barnesiella*, *Gloebacter*, *Odoribacter*, *Parabacteroides* ↓ *Eubacterium*	[74]
Luteolin	Rats	12 weeks	0.5% Luteolin	↑ *Parvibacter*, *Faecalitalea*, *Allobaculum* sp., *Bacteroides dorei*	[75]
Myricetin	Mice	12 weeks	0.5% Myricetin	↑ *Allobaculum*, *Lactobacillus*, *Nocardiaceae Tyzzerella 4*, *Brachybacterium*	[76]
Procyanidins	Obese male mice	12 weeks	100 mg/kg	↑ *Bacteroidetes* ↓ *Firmicutes/Bacteroidetes*	[77]
Quercetin	Low-density lipoprotein receptor-null mice	8 weeks	100 μg/day	↑ *Actinobacteria*, *Bacteroidetes*, *Akkermansia*, *Bacteroides*, *Parabacteroides*, *Ruminococcus*, ↓ *Firmicutes*, *Lactobacillus*	[78]
Resveratrol	Diabetic male rats	28 days	20 mg/kg	Increase in the richness indices of gut microbiota, Shaped composition of gut microbiota (e.g., increased beta diversity of the gut microbiota community), Impact on the metabolism of gut microbiota (amino acid and lipid metabolism) and defensive mechanisms	[79]
Rosmarinic acid	Diabetic rats	8 weeks	30 mg/kg	Prebiotic effect on gut microbiota	[80]
Vanillin	Obese mice	12 weeks	0.1% Vanillin	↓ *Firmicutes phylum*, ↑ *Bacteroidetes*, *Verrucomicrobiota phyla*	[81]

↑ increased compared to control; ↓ decreased compared to control.

**Table 2:** Effect of excessive body weight on the structure of the gut microbiota.

Experimental Factor	Effects on Gut Microbiome	Ref.
Normal weight *n* = 10 Obese *n* = 10	↑ *Firmicutes* ↑ *Fusobacteriums*	[140]
Normal weight *n* = 25 Obese *n* = 17 Obese plus metabolic syndrome *n* = 25	↑ *Firmicutes*	[141]
Normal weight *n* = 138 Overweight *n* = 171 Obese *n* = 132	↓ *Bakteroidetes* ↓ *Bacteroidetes/Firmicutes*	[142,143]
Normal weight *n* = 261 Overweight *n* = 170 Obese *n* = 58	↑ *Bacteroidetes* *↑ Fusobacterium* *↑ Proteobacteri*	[144]
Normal weight *n* = 28 Overweight *n* = 24	↓ *Fusobacterium*	[145]
Normal weight *n* = 23 Obese *n* = 33	↓ *Bacteroidetes* ↑ *Firmicutes/Bacteroidetes*	[146]
Normal weight *n* = 217 Overweight *n* = 163 Obese *n* = 167	↑ *Bacteroidetes*	[147]
Normal weight = 30 Obese *n* = 106	↑ *Bacteroidetes*	[148]
Normal weight *n* = 209 Overweight *n* = 563 Obese *n* = 229	↓ *Bacteroidetes* ↑ *Firmicutes*	[149]
Normal weight *n* = 11 Obese and Overweight *n* = 11	↑ *Firmicute* ↑ *Firmicutes*/*Bacteroidetes*	[150]
France: Normal weight *n* = 16; Obese *n* = 12 Saudi Arabia: Normal weight *n* = 9; Obese *n* = 9	France: ↑ *Bacteroidetes*, ↑ *Proteobacteria* Saudi Arabia: ↑ *Firmicutes*	[151]

↑ increased compared to normal body weight; ↓ decreased compared to normal body weight.

## Data Availability

Data sharing is not applicable.
